# Resilience and Parental Burnout Among Finnish Parents During the COVID-19 Pandemic: Variable and Person-Oriented Approaches

**DOI:** 10.1177/10664807211027307

**Published:** 2022-04

**Authors:** Matilda Sorkkila, Kaisa Aunola

**Affiliations:** 1 Department of Psychology, 4168 University of Jyväskylä, Jyväskylä, Finland

**Keywords:** parental exhaustion, socially prescribed perfectionism, structural equation modeling, latent profile analysis

## Abstract

During the coronavirus disease 2019 (COVID-19) crisis, different personality characteristics may have influenced parental well-being in different ways. In the present study, we combined variable and person-oriented approaches and examined relationships between resilience, parental burnout, and perfectionism during the lockdown. We first used structural equation modeling to assess the paths between variables. We then used latent profile analysis to examine different profiles of parents based on resilience, perfectionism, and symptoms of parental burnout. Finally, we examined how these profiles differ in terms of relevant background variables. The results showed that resilience predicted parental burnout negatively even after controlling for multidimensional perfectionism. Parents’ age, children's age, children's special needs, and the increase in time spent with children due to lockdown contributed independently to burning out as a parent. Three profiles were found: a resilient profile, perfectionist profile, and burned-out profile. Resilient parents were likely to be men, older, and with less financial difficulties than parents in the other two profiles, and less likely to spend increased time with their children due to lockdown than the burned-out parents. Perfectionist parents, in turn, had older children than the burned-out parents did. These results suggest that resilience may help parents overcome burnout at times of crisis.

In the Spring of 2020, families across the world were under lockdown due to the novel coronavirus disease 2019 (COVID-19). To avoid infection, people were instructed to avoid social contacts and stay home (Finnish Ministry of Health and Welfare, 2020). Many parents had to work remotely, homeschool their children, and take care of the home chores simultaneously. Due to recommendations of social distancing, grandparents or other natural source of support were unavailable and leisure activities were shut down. Due to these increased demands and decreased resources, many parents were likely to be at increased risk for parental burnout (Griffith, 2020). However, as personality factors are central predictors of parental burnout (Mikolajczak et al., 2018b), the ongoing crisis may have impacted different parents in different ways. For example, those parents who were resilient—that is, able to bounce back from hardships easily—may have been able to cope with the crisis relatively well, whereas parents with low resilience may have been at risk of burnout. In the present study, we combined variable- and person-oriented approaches and examined, first, how resilience, perfectionism, and relevant background variables are associated with parental burnout among Finnish parents during COVID-19 lockdown. Second, we assessed what kind of different profiles, based on these personality characteristics and parental burnout symptoms, exist among the parents, and how relevant background variables are associated with different profiles.

## Parental Burnout

Parental burnout has been defined as a stress-related syndrome that consist of emotional exhaustion as a parent (i.e., chronic fatigue that does not go away by resting), being fed up as a parent (i.e., not enjoying parenting anymore), emotional distancing from children (i.e., parent is able to perform only the instrumental aspects of parenting but the warmth disappears), and contrast in previous parental self (i.e., parent feels no longer as good parent as they once were) (Roskam et al., 2018). Parental burnout is often accompanied by feelings of guilt and shame (Roskam et al., 2018) and many parents report feeling trapped in an adverse situation with no way out (Hubert & Aujoulat, 2018). Consequently, it is not surprising that parents with burnout may experience substance abuse, escapism, and suicidal thoughts (Mikolajczak et al., 2018a). Furthermore, parental burnout has a very specific effect on neglect and violence toward children (Mikolajczak et al., 2018a).

Parental burnout has been suggested to develop as a result of chronic parenting-related stress, where the demands constantly exceed the available resources of the parents (Mikolajczak & Roskam, 2018). The demands are stress-producing factors (e.g., housework overload, high parental demands) and the resources are stress-alleviating factors (e.g., emotional support, self-compassion as a parent). In the model of resources and demands (BR^2^; Mikolajczak & Roskam, 2018), the central theme is the balance: even if one faces many demands but has equally many resources, s/he may avoid burning out. If there are only few demands, but even fewer resources, one may nevertheless burn out.

During the COVID-19 crisis, the demands of many parents have increased significantly, whereas the resources, in turn, have decreased (Griffith, 2020). Parents in lockdown had to work, homeschool their children, take care of the household chores at the same time (i.e., increased demands). At the same time, due to social distancing, meeting family (e.g., grandparents) and friends was prohibited, and most hobby activities were temporarily shut down (i.e., decreased resources). Furthermore, many businesses reduced services or were shut down, generating financial difficulties and unemployment, which both have been associated with parental burnout (Sorkkila & Aunola, 2020). It, consequently, makes sense to expect that during the COVID-19 crisis many parents would have been at risk of burning out.

## Resilience

Although during the COVID-19 crisis many parents were similarly under a multitude of demands, not all parents burned out (see Sorkkila, 2020). One factor that may have distinguished those parents from those who were burned out is resilience. Resilience refers to be the ability to bounce back or recover from stress (Smith et al., 2008). Resilience may buffer against a vast range of adversities (e.g., from mild everyday hassles to major traumatic events) and it may explain why some people can survive—or even thrive on—the difficult experiences of life (Fletcher & Sarkar, 2013). In general, resilience has been considered a relatively stable personality characteristic—a constellation of psychological flexibility and general resourcefulness—which enables the individual to adapt well to encountered situations (Fletcher & Sarkar, 2013, 2014). However, there is also evidence suggesting that resilience may change over time, and is therefore a dynamic process rather than stable, which can be learned (Luthar et al., 2000).

In the previous literature, resilience has been positively associated with active coping, optimism, and positive reframing, and negatively with anxiety, depression, pessimism, and self-blame (Windle, 2011). In parenting context, resilience has been associated with adaptive parenting styles characterized by acceptance and involvement (Zakeri et al., 2010) and resilience-based intervention programs have been used to strengthen parent and child competencies and reduce children's conduct problems (Borden et al., 2010). Although to the best of our knowledge the relationship between parental burnout and resilience has not yet been investigated, in other contexts it has been shown that resilience protects individuals from burning out. For example, it has been shown that student-athletes with high resilience are less likely to burn out in sport and school (Sorkkila et al., 2019) and that resilient health care professionals are less likely to burn out at work than those with lower resilience (for a review, see McCann et al., 2013). It is, consequently, also presumable that those parents who are resilient—and can thus face more flexibly the everyday adversities of parenting—are less likely to burn out.

## Background Variables

In the previous literature, several background variables have been associated with parental burnout, such as being a young parent, being a mother versus father, being unemployed or with financial difficulties, or having a child with special needs (Sorkkila & Aunola, 2020). Nevertheless, it has been shown that background variables may play a relatively small role inf parental burnout, and personality factors play a larger role (Mikolajczak et al., 2018b, Sorkkila and Aunola, 2020). Among Finnish parents, socially prescribed perfectionism (SPP)—that is, the perception that other people are setting overly high demands for the individual (Hewitt & Flett, 1991)—has been shown to be a strong risk factor for parental burnout, effect of which is even stronger if the individual also has high demands toward oneself (i.e., high on self-oriented perfectionism [SOP]; Sorkkila and Aunola, 2020). Consequently, studies that assess personality factors of parental burnout should take into account the two dimensions of perfectionism.

During COVID-19 lockdown some background variables may play a larger role than during “normal times.” For example, although in previous studies the number of children did not independently predict parental burnout (e.g., Mikolajczak et al., 2018; Sorkkila and Aunola, 2020), during lockdown having many children may have had a stronger effect due to cumulative demands (taking care of many children and their homework takes simply more time and effort). Furthermore, families had no support from the grandparents or school/preschool. Following the same logic, during lockdown having young children would be expected to produce more demands due to constant attention and care. It is, finally, possible that parents who were living in the countryside (compared to cities) and who had a yard where the children could play, would be less burned out as parents due to more space between family members.

## The Present Study

The present study aimed to assess, for the first time, how resilience, on the one hand, and how relevant background variables, on the other hand, are associated with parental burnout during the COVID-19 lockdown. As multidimensional perfectionism has been shown to play a significant role in parental burnout (Sorkkila & Aunola, 2020), its effect was taken into account. To gain a holistic understanding of the novel phenomenon, we combined a variable-oriented approach (e.g., the population is assumed to be homogeneous) with a person-oriented approach (e.g., the population is assumed to be heterogeneous). For example, during the COVID-19 crisis, there may have been distinct profiles of parents based on burnout symptoms, resilience, and multidimensional perfectionism. To identify different profiles will help targeting support more accurately at times of future lockdowns.

In the present study, we sought to answer the following research questions:

1. How do resilience and multidimensional perfectionism (socially prescribed, SOP), on the one hand, and background variables (gender, parents’ age, childrens’ age, the number of children, families’ financial situation, employment, having children with special needs, the time spent with children daily, the change in time spent with children daily, living area of the family) on the other hand, predict parental burnout during COVID-19 lockdown?
2. Can distinct profiles of parents be identified based on symptoms of parental burnout, resilience, and multidimensional perfectionism?
3. Do these profiles differ based on the relevant background variables?

## Method

### Participants

The participants of the present study were 1,105 Finnish parents (88% mothers). A total of 97% of the parents reported their family being isolated to their homes due to the COVID-19 crisis. The mothers' age varied between 19 and 59 (*M* = 38.1; *SD* = 6.6) and the fathers' age varied between 26 and 60 (*M* = 41.1; *SD* = 7.5). On average, the parents had two children (*M* = 2.3; *SD* = 1.6). The age distribution of the children is shown in Table 1. Out of the parents, 64% were employed, 19% had a job but they were not working at the moment, and 17% had no paid professional activity. A total of 65% of the employed parents reported working remotely from home during the COVID-19 crisis. Majority of the parents (43%) described their income as average, nearly one-third (36%) described their income as good, and nearly one-fifth (21%) described their income as lower than average. A total of 44% of the parents had a higher University degree, 6% had a lower University degree, 26% had a technical college degree, 22% had a vocational school degree, and 3% had no vocational degree. A total of 74% of the parents lived in a nuclear family, 11% lived in a single-parent household, and 10% lived in a blended family. Although the sample was not representative of all Finnish parents, the sample represented the Finnish parents relatively well in terms of family type and education (Official Statistics of Finland, 2021a). The average amount of children in the present study was somewhat higher than in the Finnish population (on average 1.85 children; Official Statistics of Finland, 2021b).

**Table 1.** Childrens' Age Distribution.

	Has one child in this category	Has more than one child in this category	Has no children in this category
*Children*’*s age in years*			
Less than 1	12.6	0.1	87.3
1–4	35.4	10.7	53.9
5–9	32.6	16.8	50.6
10–14	3.8	1.7	94.5
15–18	12.0	4.7	83.3
19 or older	3.8	1.7	94.5

### Procedure

The data was gathered from April 22 to May 13, 2020, which was the time in Finland when the Government declared a state of emergency in Finland due to the coronavirus outbreak (declared on March 16; Finnish Ministry of Social Affairs and Health, 2020). During this time, schools and educational institutions were closed down and contact teaching was suspended. Public gatherings were limited to no more than 10 people, and it was recommended to avoid spending any time in public places. All national museums, theaters, cultural venues, libraries, sports facilities, and hobby and leisure activities were closed. Religious communities were advised to do the same. Visits to housing services for the elderly and other risk groups were prohibited, and persons over 70 years were instructed to refrain from contact with other people. The Finnish borders were closed, and Finnish citizens were instructed not to travel abroad (Finnish Ministry of Social Affairs and Health, 2020).

An ethical approval for the study was permitted from the relevant university. Prior to participation, all participants filled out an informed consent to confirm their voluntary participation in the study. The participants filled an online questionnaire on a given Webropol-link that was advertised on university websites and different sites of social media. The answers were transmitted into IBM SPSS Statistical program (version 24).

### Measures

*Parental Burnout.* Parental burnout was measured using the Parental Burnout Assessment (Roskam et al., 2018) which has been translated to Finnish and validated in Finland by the authors (Aunola et al., 2020). The scale consists of 23 items: nine measure exhaustion in the parental role (e.g., *I feel completely run down by my role as a parent*), six measure contrast in the parental self (e.g., *I don*’*t think I*’*m the good father/mother that I used to be to my children*), five measure feelings of being fed up as a parent (e.g., *I can*’*t stand my role as father/mother anymore*), and three measure emotional distancing from one's children (e.g., *I do what I*’*m supposed to do for my children but nothing more*). All items were rated on a 7-point Likert scale indicating how often the parent feels a certain way (0 = never; 6 = every day). The Cronbach alpha reliabilities for the subscales were 0.95, 0.93, 0.90, and 0.74, respectively.

*Resilience.* Resilience was measured using the Brief Resilience Scale (Smith et al., 2008) which was translated to Finnish by the authors. The scale consists of four items which measure the ability to recover after stress (e.g., *I tend to bounce back quickly after hard times*) which are rated on a 5-point Likert scale (1 = *strongly disagree*; 5 = *strongly agree*). The Cronbach alpha reliability for the scale was 0.85.

*Background Variables.* Parents were asked about their gender, age, number of children, age of each children (the number of children in each age category “1–4,” “5–9,” “10–14,” “15–18,” “19 or older”), unemployment (“yes”/”no”), level of education (1 = no vocational education, 2 = vocational school degree, 3 = vocational institution degree; 4 = lower technical college degree, 5 = higher technical college degree, 6 = lower University degree, 7 = higher University degree), perceived family income (1 = excellent, 2 = higher than average, 3 = average, 4 = poorer than average, 5 = poor), developmental delays or special needs of any child (1 = yes, one child, 2 = yes, more than one child, 3 = no), the number of hours spent with one's children, the number of hours that one used to spend before the coronavirus crisis, the living area of the families (a) Countryside, (b) Conurbation, (c) Suburb, (d) City, and, finally, if they have a house yard or a garden where the children can play and spend time during isolation (“yes”/”no”). To form a variable describing the change in time spent with children due to COVID-19, the previous hours were decreased from the current hours.

### Analysis Strategy

All analyses were conducted using the M-plus statistical package (Muthén & Muthén, 2017).

*A Variable-Oriented Approach.* The analyses were carried out using structural equation modelling (SEM). First, measurement models for two latent variables, that is, parental burnout and resilience, were tested. The four subscales of parental burnout were used as indicators of latent parental burnout. The indicators of latent resilience, in turn, were the four observed items used to measure resilience.

Second, background variables were included in the previous model and paths from them to both latent burnout and latent resilience variables were estimated. The residuals of latent burnout and resilience were allowed to correlate. At the final stage of the modelling, nonsignificant variables were excluded from the final model. Due to large sample size, the significance level of 0.01 was used.

Third, the role of resilience in parental burnout was tested by adding a path from latent resilience to latent parental burnout. Fourth, to test whether the results would remain the same after controlling for the impacts of SOP and SPP (Sorkkila and Aunola, 2020), measurement models of SOP and SPP were included into the model. Paths from SOP and SPP to parental burnout were estimated. In the model, SOP, SPP, and resilience were allowed to correlate. Similarly, SOP and SPP were allowed to correlate with the background variables.

The parameters of the SEM models were estimated using the full-information maximum likelihood ratio procedure. Goodness of fit was evaluated using three indicators: The goodness of fit was evaluated with the following four indices: (a) Bentler's (1990) comparative fit index (CFI), (b) the Tucker–Lewis index (TLI), (c) the root mean square error of approximation (RMSEA), and (d) the standardized root mean square residual (SRMR). The fit of the model was considered to be acceptable when the CFI and TLI were 0.90 or above and the values of RMSEA and SRMR were 0.08 or below (see Hu & Bentler, 1999; Marsh et al., 2005).

*Person-Oriented Approach.* In the second stage of analyses, latent profile analysis (LPA) was applied to identify homogenous subgroups of parents (i.e., different profiles) based on the four latent variables: parental burnout, resilience, SOP, and SPP. First, LPA was estimated separately from one- to seven-class solutions. To ensure the validity of each class solution, several different starting values were used for the parameters (Muthén & Muthén, 2017). To find out the most optimal number of latent profiles, the following statistical criteria were used to compare the different solutions: (a) Akaike's information criterion (AIC); (b) the sample-size adjusted Bayesian information criterion (aBIC); (c) the Vuong–Lo–Mendel–Rubin (VLMR) test; (d) the Lo–Mendel–Rubin test (LMR); (e) the parametric bootstrapped likelihood ratio test (BLRT; Muthén & Muthén, 2017); and (f) the reliability of classification by entropy. The model with lower absolute value of the Log L, AIC, and aBIC was considered to demonstrate a better fit to the data. The likelihood ratio tests (VLMR, LMR, and BLRT) compare solutions with different numbers of latent profiles. A low *p* value (*p* < .05) suggests that a solution with one less class should be rejected in favor of the estimated model. The entropy assesses the statistical quality of the classification, that is, the precision with which the cases are classified into the different latent profiles: the larger the value and the closer it is to 1, the less there is classification error in the model. In addition to the statistical criteria, class sizes, and theoretical interpretation of the classes were taken into account while choosing the final model.

Second, background variables were included in the model as auxiliary indicator variables in line with the auxiliary measurement-error-weighted method (BCH; [Bibr bibr101-10664807211027307]Asparouhov & Muthén, 2014). By applying the method, the differences between the latent profile groups in external variables (i.e., auxiliary variables) are tested with a Chi-square test, without letting these external variables affect the formation of the latent profiles.

## Results

### Variable-Oriented Approach

*Measurement Model.* The measurement model of latent parental burnout and latent resilience constructs fit the data well: χ^2^(19) = 123.786, *p* < .001; CFI = 0.975; TLI = 0.964; RMSEA = 0.071; and SRMR = 0.042. However, an inspection of modification indices revealed that the two positively worded items of resilience had a relatively high residual correlation (0.49). Consequently, this residual correlation was estimated to end up to the final model (χ^2^(18) = 81.499, *p* < .001; CFI = 0.985; TLI = 0.977; RMSEA = 0.057; SRMR = 0.032).

**Figure 1.**
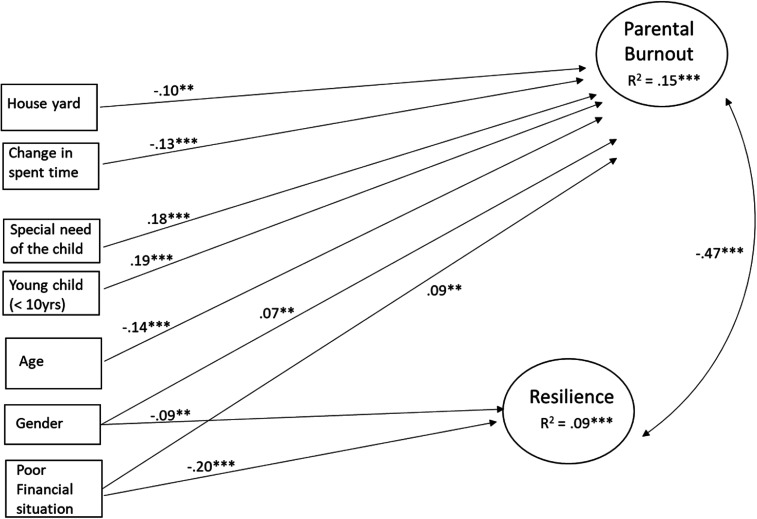
Background variables as predictors of parental burnout and resilience (structural equation model). Only statistically significant paths on the level *p *< .01 are presented. ***p < .001, **p < .01.

*Background Variables as Predictors.* Next, the role of different family background variables on parental burnout and resilience was tested adding background variables into the model as predictor variables. From the 11 tested background variables, seven were found to be statistically significantly associated with parental burnout, resilience, or both. Four variables (i.e., living in the countryside, parental unemployment, parental education, and number of children at home), in turn, were not associated with either of the dependent variables and, consequently, were excluded from the final model. After this specification, the fit of the model was: χ^2^(60) = 173.040, *p* <.001; CFI = 0.979; TLI = 0.971; RMSEA = 0.042; SRMR = 0.027. The final model is presented in Figure 1 (only statistically significant paths are shown in the figure).

The results (Figure 1) showed first that from the background variables the strongest predictors of parental burnout were having children with special needs and having children under 10 years old. The results showed further that the poorer the financial situation of the family, the younger the parent, and the more the time spent with children due to COVID-19 situation, the higher the level of parental burnout the parents reported. Finally, mothers reported a higher level of burnout than fathers and those who did not have outdoor possibility reported more burnout than those who had that possibility.

Second, two of the background variables were associated with the level of resilience: the better the financial situation of the family, the more resilience she/he reported. Moreover, fathers reported more resilience than mothers.

The background variables explained a total of 15% of the variation in parental burnout (*p* < .001) and 9% (*p* < .001) of variation in resilience.

**Figure 2.**
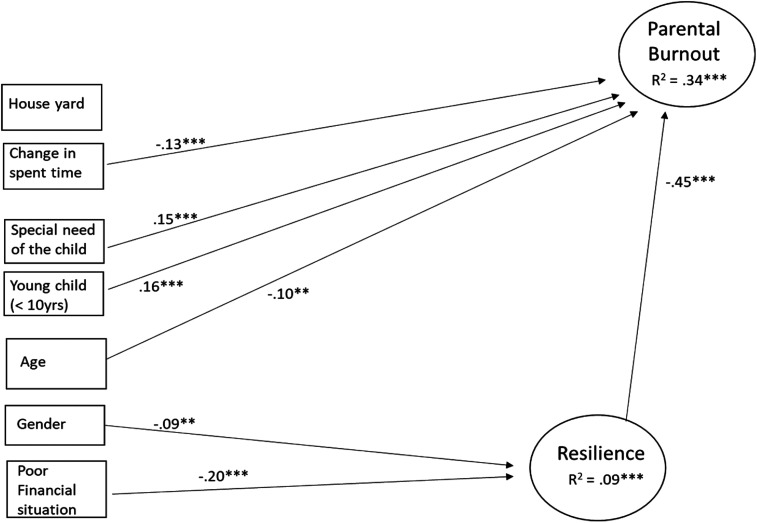
Background variables and resilience as predictors of parental burnout (structural equation model). Only statistically significant paths on the level *p *< .01 are presented. ***p < .001, **p < .01.

*Resilience as a Predictor of Parental Burnout*. Next, the role of resilience in parental burnout was tested by including the related path to the model. The results showed that resilience was statistically significantly and negatively associated with burnout: the higher the level of resilience the parent showed, the lower his/her level of parental burnout. After including the path from resilience to burnout into the model, the existing outdoor, financial situation of the family, and parent’s gender were not anymore directly associated with burnout (suggesting that they impact on burnout are rather indirect via resilience than direct). Resilience explained 19% of the variation in parental burnout. The results are shown in Figure 2.

Finally, as mentioned in the previous study it was found that SOP and SPP are important predictors of profile analysis (PA), latent variables of these were included in the model. The model fit the data well: χ^2^(159) = 603.093, *p* < .001; CFI = 0.950; TLI = 0.934; RMSEA = 0.050; SRMR = 0.041.

The results showed that even after controlling for the impacts of SOP and SPP, resilience was associated with PA (standardized beta = −0.39, *p* < .001). From perfectionism variables, only SPP predicted PA (standardized beta = 0.17, *p* < .001): the higher the SPP, the higher the level of parental burnout.

### Person-oriented Approach

The model fit indices and group sizes of two- to six-profile solutions of LPA are shown in Table 2. The AIC and aBIC values decreased when the number of profiles increased (see Table 2), suggesting that even more than six profiles could be found. Similarly, the BLRT suggested that even more than six profiles could be identified. However, according to the VLMR and the LMR results, the three-profile solution was better than the two-profile solution and increasing the number of profiles did not improve the fit of the model. Due to the entropy value being higher in the three-profile solution than in the four-profile solution as well, this solution was selected for further analysis.

**Table 2.** Model Fit Indices for Solutions With Different Number of Latent Profiles (*N* = 1,105).

	aBIC	AIC	Entropy	VLMR (*p*)	LMR (*p*)	BLRT (*p*)	Sample sizes
2 profiles	42,058.82	41,991.06	0.955	<.001	<.001	<.001	212, 893
3 profiles	40,188.08	40,111.16	0.903	<.001	<.001	<.001	160, 352, 593
4 profiles	39,888.00	39,801.93	0.813	.454	.464	<.001	152, 227, 296, 430
5 profiles	39,004.69	38,909.46	0.911	.436	.442	<.001	86, 88, 130, 261, 540
6 profiles	38,732.93	38,628.545	0.852	.175	.178	<.001	68, 72, 137, 185, 261, 382

*Note.* AIC = Akaike's information criterion; aBIC = sample size adjusted Bayesian information criterion; LMR = Lo–Mendell–Rubin adjusted likelihood test, *p* value; VLMR = Vuong–Lo–Mendell–Rubin likelihood ratio test, *p* value; BLRT = Bootstrapped Likelihood Ratio Test, *p* value.

**Figure 3.**
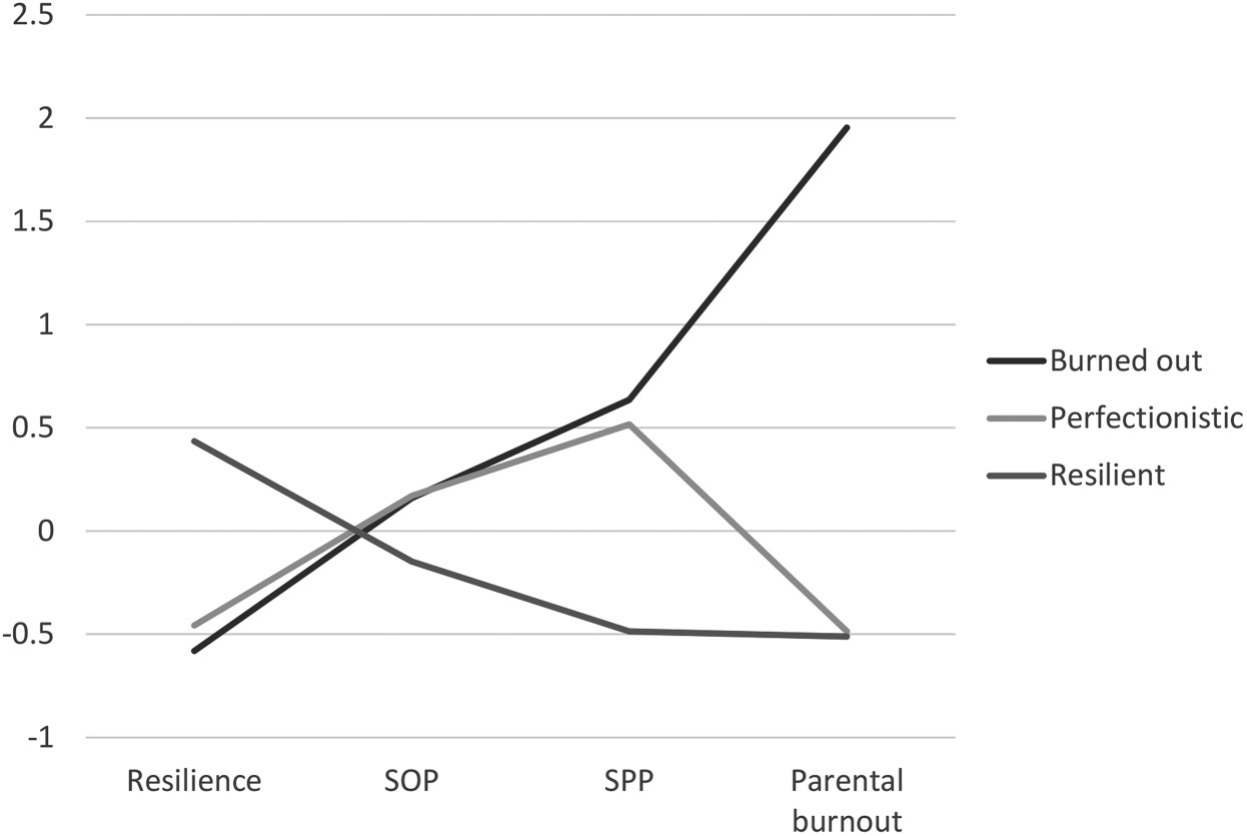
Latent profiles of parents based on resilience, SOP, SPP, and parental burnout. *Note*. SOP, self-oriented perfectionism; SPP, socially prescribed perfectionism.

The three identified latent profiles are shown in Figure 3. About half of the sample (54%) showed a profile characterized by high level of resilience, low levels of both types of perfectionism, and low level of parental burnout. The group was labeled as *Resilient* parents. The second and the smallest group (14%) was profile characterized by an opposite pattern of characteristics, that is low level of resilience, high perfectionisms, and substantially high level of parental burnout compared to other groups. This group was labeled as *Burned-out* parents. The third group consisted one-third of the sample (32%) and was characterized as low level of resilience and as a high level of SPP and SOP than the previous group, but the lower level of parental burnout. This group was labeled as *perfectionistic* parents.

Next, the differences between the three profiles in background variables were investigated. The results of these analyses are shown in Table 3.

**Table 3.** Group Means (*M*) and *SD* of Background Variables in Different Latent Profile Groups and Pairwise Comparisons Between the Groups.

	Group 1 burned out	Group 2 perfectionist	Group 3 resilient	χ^2^	3
	*M* (*SE*)	*M* (*SE*)	*M* (*SE*)		
Parent’s gender	1.937 (0.021)^a^	1.914 (0.016)^a^	1.850 (0.015)^b^	11.611	0.003
Parent’s age	36.278 (0.456)^a^	37.593 (0.368)^a^	39.567 (0.295)^b^	36.046	<0.001
Financial situation	3.002 (0.083)^a^	2.964 (0.051)^a^	2.660 (0.038)^b^	25.606	<0.001
Under 10 years at home	0.947 (0.019)^a^	0.811 (0.022)^b^	0.754 (0.018)^b^	48.874	<0.001
Special need	1.392 (0.040)^a^	1.278 (0.025)^ab^	1.215 (0.017)^b^	15.872	<0.001
Change in used time	−4.620 (0.274)^a^	−3.787 (0.193)^ab^	−3.533 (0.141)^b^	11.487	0.003
Outdoor	1.760 (0.035)	1.792 (0.022)	1.849 (0.015)	6.774	0.034

*Note.* Means with the same superscripts do not differ significantly from each other.

The results showed statistically significant group differences in six of the seven tested variables (see Table 3). The Resilient parents were on average older and had better financial situation at family than those in the other two groups. Moreover, the number of fathers was higher in this group than in other two groups. Parents showing the Burned-out profile were more likely to have under 10-year old child/ren or child/ren with special needs, and they were also more likely to spend increased time with their children due to the COVID-19 lockdown than the parents in the resilient group. Finally, the perfectionistic parents differed from the burned-out parents in the age of children: young children were more typical for burned-out parents than for the perfectionistic parents.

## Discussion

The present study used variable- and person-oriented approaches to examine the role of resilience, perfectionism, and background variables on parental burnout during the COVID-19 lockdown. The results showed that resilience predicted parental burnout negatively: The more resilient the parents were, the less burned out they were. The results are in line with the previous findings conducted in other contexts: for example, resilience has been shown to predict negatively sport burnout (Sorkkila et al., 2019), and occupational burnout (McCann et al., 2013). Therefore, it is not surprising that resilient parents—who are likely to recover quickly from stressful situations—also become less burned out during COVID-19 lockdown.

The impact of resilience on parental burnout remained significant even when multidimensional perfectionism was added to the model. In line with previous studies, SPP had a unique contribution to parental burnout (Sorkkila & Aunola, 2020), indicating that high expectations from other people enhance burning out. Out of the background variables, being a young parent, having young children, having children with special needs, and spending more time with children, were significant predictors of parental burnout. Before the COVID-19 crisis, it has been similarly shown that being a young parent and having children with special needs predict parental burnout (Kawamoto et al., 2018; Sorkkila & Aunola, 2020). However, having young children and spending an increased amount of time with children may be particular risk factors during lockdown: During lockdown, children have not been able to participate in daycare, and social and practical support has been absent.

Three distinct profiles of parents were identified: resilient parents, perfectionist parents, and burned-out parents. Resilient parents were the largest group and characterized by high levels of resilience and low levels of burnout and both types of perfectionism. The second-largest group was the perfectionist parents, who showed relatively high levels of both types of perfectionism and low levels of resilience and parental burnout symptoms. The smallest group was a group of Burned-out parents, who showed the opposite pattern to the Resilient parents: they were high on both types of perfectionism, high on burnout symptoms, and low on resilience. The Resilient parents were more likely to be older, men, and with less perceived financial difficulties than those in the other two groups. As resilience can be learned (Luthar et al., 2000), it is possible that older parents have built more resilience due to more life experiences. Having fewer financial concerns may, in turn, generate less cumulative stressors and ability to buy help with household chores or childcare. As parents in the Burned-out group were more likely to have young children and children with special needs than the Resilient parents, as well as spend increased time with their children during the COVID-19 epidemic, it is not surprising, that they were more at risk of burnout. The third profile consisted of perfectionist parents, who still were not burned out. This is an interesting profile, as in previous studies perfectionism—particularly SPP—has been shown to be a strong predictor of burning out (Sorkkila & Aunola, 2020; see also Kawamoto et al., 2018). Parents in this group differed from burned-out parents in terms of children's age, in such that they had older children (>10 years old) than Burned out parents did. Perfectionism may thus be particularly harmful among parents of young children: when the children are young, family life is harder to control, and parenting is more intense than with older children. Consequently, parents with young children may benefit from skills of self-acceptance and compassion (Sorkkila & Aunola, 2020).

### Strengths and Limitations of the Study

To the best of our knowledge, the present study was the first one to examine the relationship between resilience and parental burnout. We conducted the study during a very specific time of COVID-19 lockdown and discovered that the background variables that impact parental burnout are very different than during normal times (see, e.g., Sorkkila & Aunola, 2020). In this study, we combined variable- and person-oriented approaches, which allowed multifaced gathering of information that can be used in targeted interventions.

The study had, however, several limitations that need to be taken into account. First, the data was collected only at one time point, and therefore, it is not possible to draw any conclusion about directionality between the variables. Second, mothers were overrepresented in the present study, and, therefore, the results describe mainly viewpoints of mothers. Third, this sample was conducted in only one cultural context, that is, Finland. Although lockdowns due to COVID-19 virus were conducted throughout the world, the restrictions may have varied somewhat between the countries. In the future, it is important to examine the well-being of parents in various cultural contexts.

### Conclusion

This study examined, for the first time, the relationship between parental burnout and resilience, and showed that resilience may be a key character that distinguishes parents who burn out during COVID-19 lockdown from those who do not. It may be thus helpful to teach parents, particularly mothers, resilient-related skills, such as cognitive reframing and optimism. As the burned-out parents were mainly mothers who spent increased time with their young children, mental skills support should be accompanied by practical support (e.g., help with childcare and household chores). Furthermore, as perfectionism seems to be harmful to particularly mothers of young children, these skills could be accompanied by self-acceptance and self-compassion.
